# Proline-Catalysed Amination Reactions in Cyclic Carbonate Solvents

**DOI:** 10.3390/molecules16043420

**Published:** 2011-04-21

**Authors:** Christopher Beattie, Michael North, Pedro Villuendas

**Affiliations:** School of Chemistry, Research Centre in Catalysis and Intensified Processing, Bedson Building, University of Newcastle, Newcastle upon Tyne, NE1 7RU, UK

**Keywords:** proline, amination, cyclic-carbonate

## Abstract

Propylene carbonate is shown to be an environmentally friendly and sustainable replacement for dichloromethane and acetonitrile in proline-catalysed α-hydrazinations of aldehydes and ketones. Enantioselectivities comparable to those obtained in conventional solvents or ionic liquids can be obtained, even when using a lower catalyst loading.

## 1. Introduction

Proline (**1**) is unique amongst the proteinogenic amino acids in that it is a secondary amine. This leads to it having a special role in peptides and proteins as it cannot act as a hydrogen-bond donor, but is often found in β-turns [1,2,3]. Proline and its derivatives have also found many applications in synthetic chemistry as chiral-auxiliaries [4], chiral reagents [5,6] and most recently as chiral catalysts [7,8,9,10,11,12] where it was developments in proline-catalysed enamine chemistry that sparked the recent resurgence of interest in asymmetric organocatalysis [13,14,15,16,17]. Proline is undoubtedly the most sustainable of the organocatalysts as it is directly available from biological sources without any need for chemical transformations. Proline-catalysed reactions are however, commonly carried out in traditional solvents such as DMSO, DMF and chlorinated solvents [7,8,9,10,11,12] which severely undermine the green credentials of proline-catalysed reactions due to their toxicity. Although both water [18,19] and ionic liquids [20] have also been used as solvents for proline-catalysed reactions, the green credentials of both of these solvents have been questioned [21,22] and water has been shown to inhibit proline-catalysed aldol reactions [23]. In some cases, proline-catalysed reactions can also be carried out without a solvent [24,25]. This limited solvent compatibility of proline-catalysed reactions is one of the reasons that so much effort has been put into developing other asymmetric organocatalysts which are soluble in a broader range of solvents. However, we have adopted a different approach, namely the use of alternative, sustainable and non-toxic polar-aprotic solvents in proline-catalysed reactions.

**Figure 1:**
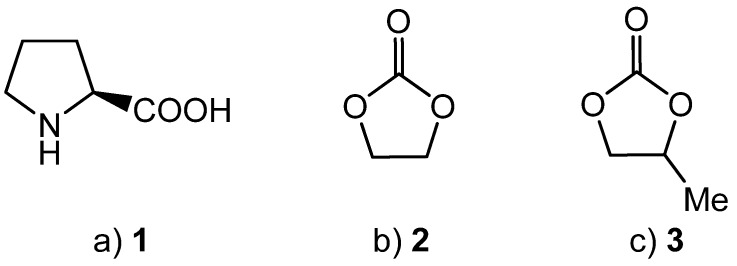
**(a)** (*S*)-Proline **1**. **(b)** Ethylene carbonate **2**. **(c)** Propylene carbonate **3**.

Cyclic carbonates (Figure 1), especially ethylene carbonate (**2**) and propylene carbonate (**3**), are attracting increasing interest as green solvents for metal-catalysed reactions [26,27,28]. Ethylene carbonate (**2**) is a solid at room temperature (m.p. 36 °C, b.p. 248 °C), though its melting point is depressed on addition of reactants. Propylene carbonate (**3**) has a very wide liquid range (m.p. −49 °C, b.p. 242 °C), making it a suitable solvent for reactions carried out below, at, or above room temperature. As a result of their high boiling points, cyclic carbonates **2** and **3** have very low vapour pressures. Compounds **2** and **3** are biodegradable, have high flash points and low odour levels [29]. They are used commercially as degreasers, paint strippers and in cleaning applications, their toxicities have been assessed and compound **3** is licensed for use in cosmetics [26]. Ethylene and propylene carbonate have high dielectric constants, so they can be considered as sustainable replacements for traditional polar aprotic solvents such as DMF, DMSO, HMPA and NMP. There is however, a significant difference in polarity between the two cyclic carbonates: ethylene carbonate **2** has a dielectric constant of 90, whilst propylene carbonate **3** has a dielectric constant of 65 [30]. Thus, the solvent polarity can be tuned by choice of the appropriate cyclic carbonate or by use of a mixture of the two solvents.

**Scheme 1:**
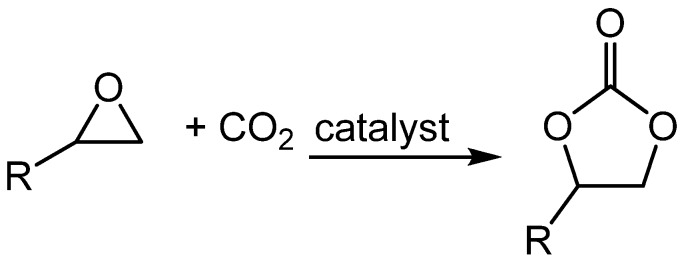
Synthesis of cyclic carbonates from epoxides and carbon dioxide.

Cyclic carbonates are prepared by the 100% atom economical reaction between carbon dioxide and ethylene or propylene oxide (Scheme 1) [31,32,33,34,35]. We have shown that, in the presence of an appropriate catalyst, the synthesis of cyclic carbonates **2** and **3** can be achieved at atmospheric pressure and room temperature for batch processes [36,37,38,39] and at temperatures of 100 °C or below in a gas phase flow reactor [40,41]. 

This opens the possibility of utilizing waste carbon dioxide from major fixed site producers such as power stations [42], oil refineries and chemical plants in the production of cyclic carbonates, especially as the catalysts have been shown to tolerate the impurities present in power station flue gas [41].

**Scheme 2:**
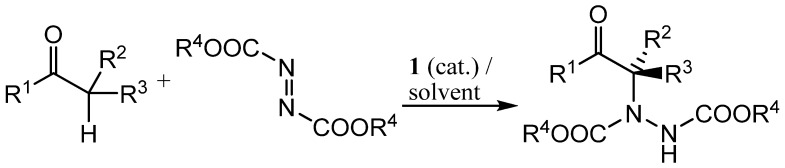
Proline-catalysed α-hydrazination of carbonyl compounds.

In recent papers we have shown that cyclic carbonates **2** and **3** make excellent solvents for proline-catalysed aldol reactions [43,44,45]. In this manuscript, we extend that study to the proline-catalysed α-hydrazination of aldehydes and ketones by diazodicarboxylates (Scheme 2). Previous work on this extensively utilized, proline-catalysed reaction has employed dichloromethane [46,47,48,49,50,51,52,53], acetonitrile [54,55,56,57,58,59,60,61,62] or an ionic liquid [63] as the solvent.

## 2. Results and Discussion

**Scheme 3:**
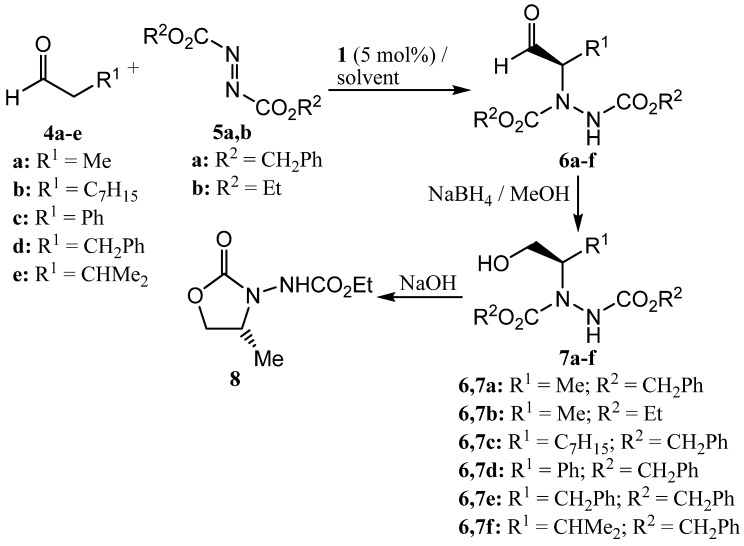
Proline-catalysed α-hydrazination of aldehydes in cyclic carbonate solvents.

For initial studies, the reaction between propanal (**4a**) and dibenzyl azodicarboxylate (**5a**) catalysed by (*S*)-proline (**1**, 5 mol%) was selected since there is literature precedent for this reaction [54] and the benzyl groups provided a convenient chromophore for our chiral HPLC system. The initially produced aldehyde **6a** was immediately reduced to the more stable alcohol **7a** by treatment with sodium borohydride (Scheme 3) and all yields and enantioselectivities refer to the formation of compound **7a**. The results are presented in Table 1. Entry 1 of Table 1 shows the result of a control experiment carried out in dichloromethane which confirmed that alcohol **7a** was obtained in excellent yield and enantioselectivity under these conditions. The absolute configuration of alcohol **7a** was determined to be *R*- on the basis of the chiral HPLC retention times [64]. 

Entries 2 and 3 of Table 1 show the results of experiments carried out in cyclic carbonates **2** and **3** under conditions which are otherwise identical to entry 1. In both cases, alcohol **7a** was obtained in good yield, but with lower enantiomeric excess than that obtained in dichloromethane. To improve the enantioselectivity of the reaction, the effect of lowering the reaction temperature was investigated. This was only possible with propylene carbonate (**3**) as solvent, and at 0 °C the reaction in propylene carbonate gave alcohol **7a** with excellent enantioselectivity, but in low yield after a standard reaction time of two hours (Table 1, entry 4). To increase the chemical yield, the reaction time was extended to 24 hours (Table 1, entry 5) and under these conditions alcohol **7a** was obtained in good yield and with excellent enantioselectivity. An attempt to use diethyl azodicarboxylate (**5b**) in place of the dibenzyl derivative was not particularly successful as alcohol **7b** could not be detected on our HPLC system, so the alcohol was allowed to cyclise to oxazolidinone **8** [46,63] which was obtained in overall 39% yield from propanal. The absolute configuration and enantiomeric excess of compound **8** could be determined by chiral GC analysis, but the latter was significantly lower than that of alcohol **7a** (Table 1, entry 6).

Taking the conditions of Table 1, entry 5 as optimal, the applicability of the chemistry to four other aldehydes was investigated (Table 1, entries 7-10). Nonanal (**4b**) gave alcohol **7c** with good enantioselectivity but only moderate yield (Table 1, entry 7). In contrast, phenylacetaldehyde (**4c**) gave alcohol **7d** in good yield, but with a low enantiomeric excess, almost certainly due to the extremely facile racemization of the initially formed aldehyde **6d** (Table 1, entry 8). Aldehydes **4d** and **4e** gave the corresponding alcohols **7e** and **7f** in both good yield and with excellent enantiomeric excesses. The enantiomeric excesses of alcohols **7a,d,e,f** compare favourably with those reported in the literature for the corresponding product prepared using 10 mol% of (*S*)-proline in dichloromethane [46] or acetonitrile [54] and in each case the (*R*)-enantiomer of the alcohol was formed predominantly [64].

**Scheme 4:**
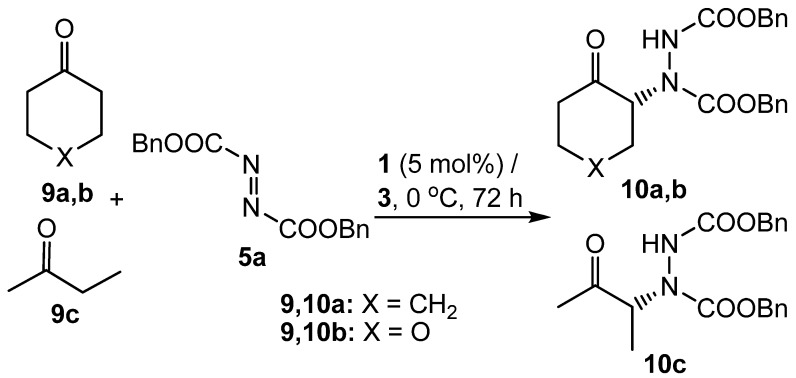
Proline-catalysed α-hydrazination of ketones in propylene carbonate.

Having demonstrated that propylene carbonate was a suitable solvent for the asymmetric α-hydrazination of aldehydes, the use of ketones as substrates was investigated. There are only two previous reports of proline-catalysed ketone hydrazination [55,63], indicating that this is a more difficult undertaking than the use of aldehydes as substrates. Cyclohexanone (**9a**) was chosen as the first test substrate and initial experiments showed that whilst reaction with dibenzyl azodicarboxylate (**5a**) did indeed occur under the standard conditions developed for aldehyde substrates, the reaction was much slower and required a reaction time of 72 hours to produce a reasonable yield of α-hydrazinoketone **10a** (Scheme 4). Compound **10a** was found to be more stable than the corresponding aldehyde adducts **6a-f** and could be isolated and characterized without the need to reduce the ketone to the corresponding alcohol. Two other ketones **9b,c** were converted into α-hydrazinoketones **10b,c** under the same conditions and the results are shown in Table 2.

Cyclohexanones **9a,b** were found to be reasonable substrates for proline catalysed α-hydrazination in propylene carbonate, giving products **10a,b** in good yield and with respectable enantiomeric excesses (Table 2, entries 1 and 2). Butanone (**9c**) was not as good a substrate, giving only a moderate yield of product **10c** and with lower enantioselectivity than that observed for the cyclohexanone derivatives (Table 2, entry 3). Notably however, this substrate did react regioselectively, as no evidence for formation of the product derived from reaction at the methyl group of ketone **9c** was observed.

In all the results discussed so far, racemic propylene carbonate has been used as solvent. We have previously shown that for proline-catalysed aldol reactions, use of enantiomerically pure propylene carbonate as solvent can affect both the yield and stereoselectivity of the transformation with matched and mismatched pair effects being seen for the stereochemistry of the catalyst and solvent [45]. Therefore, reactions involving cyclohexanone (**9a**) as substrate were carried out in enantiomerically pure (*R*)-propylene carbonate in the presence of both (*S*)- and (*R*)-proline as catalyst (Table 2, entries 4 and 5). However, in this case, no significant difference between the reactions carried out in racemic and enantiomerically pure solvent was observed, nor was there a difference between reactions carried out using the two enantiomers of proline in (*R*)-propylene carbonate.

## 3. Experimental

### 3.1. Chemicals and instrumentation

Propylene carbonate (**3**) was distilled from CaH_2_ under reduced pressure and stored over molecular sieves. Other commercially available products (Aldrich, Acros) were used as received. Chromatographic separations were performed using silica gel 60 (230–400 mesh, Davisil). Infrared spectra were recorded at room temperature on a Varian 800 FT-IR Scimitar series spectrometer. Optical rotation measurements were conducted on a Polaar 2001 Optical Activity automatic polarimeter at the sodium D-line using a 0.25 dm thermostated cell and a suitable solvent that is reported along with the concentration (g/100 mL). High- and low-resolution electrospray ionization (ES) mass spectra were recorded on a Waters LCT Premier LCMS spectrometer using direct injection of the sample in MeOH. ^1^H- and ^13^C-NMR were recorded in CDCl_3_ on a Bruker Avance 300 spectrometer at 300 and 75 MHz respectively. ^13^C-NMR spectra were also recorded at 125 MHz on a JEOL Lambda 500 spectrometer. Chiral gas chromatrogaphy was performed on a Varian450-GC instrument with a TCD detector using a Supelco Gamma DEX 120 fused silica capillary column (30 m × 0.25 mm × 0.25 µm film thickness) with hydrogen as the carrier gas (flow rate 3.5 mL/min). Analyses were performed with an initial column temperature of 165 °C, followed by a ramp rate 0.8 °C/min to 210 °C, then hold at 210 °C for 80 minutes. Chiral HPLC was performed using a Varian ProStar system comprising binary pumping modules, a diode array detector and an autosampler and equipped with a Daicel Chiralcel OD-H, AD-H or AS-H column (25 cm by 4.6 mm), using a mixture of isopropanol and hexane as eluent. The HPLC retention times of the enantiomeric products were determined using racemic samples of compounds **7a-f** and **10a-c** which were prepared from reactions catalysed by (*R,S*)-proline in dichloromethane or propylene carbonate at room temperature. 

### 3.2. Synthesis of compound ***8*** [46,63]

To a stirred solution of diethyl azodicarboxylate (**5b**, 0.18 mL, 1.00 mmol) and propanal (**4a**, 0.11 mL, 1.50 mmol) in propylene carbonate (**3**, 3 mL) was added (*S*)-proline (**1**, 5.9 mg, 0.05 mmol). The reaction was stirred at 0 °C for 24 hours, then quenched by the addition of H_2_O (5 mL), extracted with Et_2_O (20 mL), washed with H_2_O (4 × 10 mL) and dried (Na_2_SO_4_). The solvent and excess propanal were evaporated *in vacuo* and the residue was dissolved in ethanol (10 mL) and NaBH_4_ (40.0 mg, 1.05 mmol) was added. The reaction was stirred for 10 minutes at 0 °C, then aqueous ammonium chloride solution (10 mL) and EtOAc (20 mL) were added. The organic layer was separated, dried (Na_2_SO_4_) and the solvent evaporated *in vacuo*. The residue was purified by silica gel chromatography eluting with hexane:EtOAc (85:15) to give alcohol **7b**. Compound **7b** was dissolved in MeOH (2 mL) and 0.5 M aqueous NaOH (2.5 mL) and the reaction stirred for 2 hours. MeOH was then evaporated *in vacuo* and the aqueous phase extracted with EtOAc (3 × 15 mL). The combined organic layers were dried (MgSO_4_) and the solvent evaporated *in vacuo* to give compound **8** (0.91 g, 39%) as a yellow oil. ν_max_(neat) 3362 w, 3167 w, 2968 m, 2927 m, 2879 m, 1721 s and 1538 cm^−1^ m; [α]_D_^20^ -11.0 (c=0.4, CHCl_3_) (lit [65]. [α]_D_^28^ -19.9 (c=1.4, CHCl_3_)); δ_H_ 1.3–1.4 (6H, m, 2xCH_3_), 3.87 (1H, t *J* 8.4 Hz, C*H*_2_CH), 4.1–4.2 (1H, m, CHN), 4.25 (2H, q *J* 6.9 Hz, C*H*_2_CH_3_), 4.51 (1H, t *J* 8.4 Hz, C*H*_2_CH), 6.5–6.6 (1H, m, NH); δ_C_(75 MHz) 14.6 (CH_3_), 16.8 (CH_3_), 53.2 (CHN), 63.2 (CH_2_O), 69.1 (CH_2_O), 155.6 (C=O), 157.9 (C=O); Chiral GC retention times 11.1 (minor) and 12.7 (major) minutes.

### 3.3. General procedure for the synthesis of alcohols ***7***

To a stirred solution of dibenzyl azodicarboxylate (**5a**, 0.3 g, 1.0 mmol) and an aldehyde **4a–e** (1.5 mmol) in propylene carbonate (**3**, 1 mL) was added (*S*)-proline (**1**, 5.9 mg, 0.05 mmol). The reaction was stirred at 0 °C for 24 hours, then quenched by the addition of H_2_O (5 mL), extracted with Et_2_O (20 mL), washed with H_2_O (4 × 10 mL) and dried (Na_2_SO_4_). Volatiles were removed by evaporation *in vacuo* and the residue was dissolved in ethanol (10 mL) and NaBH_4_ (40.0 mg, 1.05 mmol) was added. The reaction was stirred for 10 minutes at 0 °C, then aqueous ammonium chloride solution (10 mL) and EtOAc (20 mL) were added. The organic layer was separated, dried (Na_2_SO_4_) and the solvent evaporated *in vacuo*. The residue was purified by silica gel chromatography eluting with hexane:EtOAc (85:15) to give alcohols **7a,c–f**.

*Dibenzyl (R)-1-(1-methyl-2-hydroxyethyl)hydrazine-1,2-dicarboxylate* (**7a**) [54]. Obtained as a yellow oil (0.27 g, 76%). ν_max_(neat) 3271 br, 2955 s and 1715 cm^-1^ s; [α]_D_^20^ -32.0 (c = 0.4, CHCl_3_); δ_H_ 0.85 (3H, d *J* = 6.9 Hz, CH_3_), 3.4–3.5 (2H, m, C*H*_2_OH), 4.0–4.5 (2H, m, CH and OH), 4.9–5.2 (4H, m, 2 × C*H*_2_Ph), 7.0–7.4 (10H, m, ArH); δ_C_(75 MHz) 15.5 (CH_3_), 56.4 (CH) 63.6 (CH_2_OH), 66.1(OCH_2_Ph) 68.6 (OCH_2_Ph), 128.5 (ArCH), 128.6 (ArCH), 128.9 (ArCH), 129.0 (ArCH), 135.8 (ArC), 136.3 (ArC), 157.0 (NCO_2_), 159.0 (NCO_2_); HPLC (Chiralpak AS-H using hexane: ^i^PrOH (95:5) as solvent at a flow rate of 0.5 mL/min) retention times 22.2 (major) and 24.7 (minor) minutes.

*Dibenzyl (R)-1-(1-heptyl-2-hydroxyethyl)hydrazine-1,2-dicarboxylate* (**7c**). Obtained as a colourless oil (0.18 g, 41%). ν_max_(neat) 3267 br, 2963 s and 1716 cm^-1^ s; [α]_D_^20^ -26.0 (c = 0.4, CHCl_3_); δ_H_ 0.7–0.9 (3H, m, CH_3_), 1.0–1.4 (12H, m, Me(C*H*_2_)_6_), 3.2–3.5 (2H, m, C*H*_2_OH), 4.0–4.4 (2H, m, CH and OH), 5.0–5.2 (4H, m, 2×C*H*_2_Ph), 6.90 (1H, s, N*H*), 7.0–7.4 (10H, m, ArH); δ_C_(75 MHz) 13.9 (CH_3_), 22.5 (CH_2_), 26.0 (CH_2_), 27.9 (CH_2_), 29.0 (CH_2_), 29.3 (CH_2_), 31.6 (CH_2_), 60.8 (NCH), 62.1 (CH_2_OH), 68.2 (2 × OCH_2_Ph), 127.7 (ArCH), 128.0 (ArCH), 128.1 (ArCH), 128.2 (ArCH), 128.4 (ArCH), 128.5 (ArCH), 135.3 (ArC), 135.9 (ArC), 157.4 (NCO_2_), 159.1 (NCO_2_); m/z(ES^+^) 465 (M+Na^+^, 10); Found (ES^+^) 465.2348, C_25_H_34_N_2_O_5_Na, (M+Na^+^) requires 465.2365; HPLC (Chiralpak AS-H using hexane: ^i^PrOH (90:10) as solvent at a flow rate of 0.5 mL/min) retention times 20.4 (minor) and 43.0 (major) minutes.

*Dibenzyl (R)-1-(1-phenyl-2-hydroxyethyl)hydrazine-1,2-dicarboxylate* (**7d**). Obtained as a white solid (0.29 g, 70%). m.p. 140–141 °C; ν_max_(neat) 3273 br, 2946 s and 1711 cm^-1^ s; [α]_D_^20^ -38.0 (c = 0.4, CHCl_3_); δ_H_ 3.2–3.5 (2H, m, C*H*_2_OH), 4.0–4.5 (2H, m, CH and OH), 4.8–5.1 (4H, m, 2 × C*H*_2_Ph) 7.0–7.4 (15H, m, ArH); δ_C_(75 MHz) 56.7 (CH), 63.5 (CH_2_OH), 65.5 (CH_2_Ph), 70.2 (CH_2_Ph), 127.8 (ArCH), 127.9 (ArCH), 128.2 (ArCH), 128.6 (ArCH), 129.0 (ArCH), 135.7 (ArC), 136.2 (ArC), 156.3 (NCO_2_), 159.3 (NCO_2_); HPLC (Chiralpak AD-H using hexane: ^i^PrOH (90:10) as solvent at a flow rate of 0.5 mL/min) retention times 46.2 (minor) and 48.7 (major) minutes. 

*Dibenzyl (R)-1-(1-benzyl-2-hydroxyethyl)hydrazine-1,2-dicarboxylate* (**7e**) [54]. Obtained as a white solid (0.33 g, 76%). m.p. 105–106 °C (lit [68]. 110–116 °C); ν_max_(neat) 3272 br, 2963 s and 1716 cm^−1^ s; [α]_D_^20^ +11.1 (c = 0.9, CHCl_3_) (lit [68] [α]_D_^25^ +11.4 (c = 1.8, CHCl_3_)); δ_H_ 2.4–2.7 (2H, m, C*H*_2_Ph), 3.3–3.6 (2H, m, C*H*_2_OH) 4.4–4.6 (2H, m, CH and OH), 4.9–5.2 (4H, m, 2 × OC*H*_2_Ph), 6.8–7.4 (15H, m, ArH); δ_C_(75 MHz) 34.5 (CH_2_), 60.2 (CH), 61.8 (CH_2_OH), 68.0 (OCH_2_Ph), 68.3 (OCH_2_Ph), 128.2 (ArCH), 128.5 (ArCH), 128.7 (ArCH), 128.8 (ArCH), 128.9 (ArCH), 129.0 (ArCH), 129.1 (ArCH), 129.2 (ArCH), 129.4 (ArCH), 135.1 (ArC), 135.7 (ArC), 137.3 (ArC), 156.7 (NCO_2_), 158.8 (NCO_2_); m/z(ES^+^) 443 (M+Na^+^, 50), 863 (2M+Na^+^), 1283 (3M+Na^+^); Found (ES^+^) 443.1550, C_24_H_24_N_2_O_5_Na, (M+Na^+^) requires 443.1583; HPLC (Chiralpak AS-H using hexane: ^i^PrOH (85:15) as solvent at a flow rate of 1.0 mL/min) retention times 13.4 (major) and 31.5 (minor) minutes.

*Dibenzyl (R)-1-(1-(1-methyl)ethyl-2-hydroxyethyl)hydrazine-1,2-dicarboxylate* (**7f**) [54] Obtained as a yellow oil (0.34 g, 87%). ν_max_(neat) 3272 br, 2963 s and 1716 cm^−1^ s; [α]_D_^20^ -21.0 (c = 0.4, CHCl_3_); δ_H_ 0.6–1.0 (6H, m, 2 × C*H*_3_), 1.4–1.7 (1H, m, C*H*Me_2_) 3.3–4.4 (4H, m, NC*H*C*H*_2_O*H*) 4.9–5.2 (4H, m, 2 × C*H*_2_Ph) 6.7–6.9 (1H, br s, NH) 7.1–7.4 (10H, m, ArH); δ_C_ (75 MHz) 19.3 (CH_3_), 20.0 (CH_3_), 27.5 (CH), 60.4 (CH), 67.2 (CH_2_OH), 68.4 (2 × OCH_2_Ph), 127.7 (ArCH), 128.0 (ArCH), 128.1 (ArCH), 128.2 (ArCH), 128.4 (ArCH), 128.6 (ArCH), 135.2 (ArC), 135.9 (ArC), 156.4 (NCO_2_), 157.4 (NCO_2_); HPLC (Chiralpak OD-H using hexane: ^i^PrOH (90:10) as solvent at a flow rate of 1.0 mL/min) retention times 14.4 (major) and 17.7 (minor) minutes.

### 3.4. General procedure for the synthesis of ketones ***10a-d***

To a stirred solution of dibenzyl azodicarboxylate (**5a**, 0.3 g, 1.0 mmol) and a ketone (1.5 mmol) in propylene carbonate (**3**, 1 mL), was added (*S*)-proline (**1**, 5.9 mg, 0.05 mmol). The reaction was stirred at 0 °C for 24 hours, then quenched by the addition of H_2_O (5 mL), extracted with Et_2_O (10 mL), washed with H_2_O (4 × 10 mL) and dried (Na_2_SO_4_). Volatiles were removed by evaporation *in vacuo* and the residue was purified by silica gel chromatography eluting with hexane:EtOAc (70:30) and recrystallized with cold isopropanol to give ketones **10-d**.

*Dibenzyl (R)-1-(2-oxocyclohexyl)hydrazine-1,2-dicarboxylate* (**10a**) [66]. Obtained as a colourless oil (0.28 g, 71%). ν_max_(neat) 3271 br, 2965 s, 1797 s, 1743 s and 1679 cm^−1^ m; [α]_D_^20^ -21.0 (c = 0.1, CH_2_Cl_2_) (lit [66]. [α]_D_^20^ -24.1 (c=0.1, CH_2_Cl_2_)); δ_H_ 1.6–2.5 (8H, m, (CH_2_)_4_), 5.1-5.2 (5H, m, 2 × CH_2_Ph and NCH), 6.88 (1H, br, NH), 7.3–7.5 (10H, m, ArH); δ_C_ (75 MHz) 24.4 (CH_2_), 26.7 (CH_2_), 30.8 (CH_2_), 41.2 (CH_2_), 66.5 (2 × OCH_2_Ph), 67.9 (NCH), 127.7 (ArCH), 128.0 (ArCH), 128.1 (ArCH), 128.2 (ArCH), 128.5 (2 × ArCH), 135.9 (2 × ArC), 156.3 (2 × NCO_2_), 206.9 (CO); HPLC (Chiralpak OD-H using hexane: ^i^PrOH (90:10) as solvent at a flow rate of 0.9 mL/min) retention times 16.9 (minor) and 22.6 (major) minutes.

*Dibenzyl (R)-1-(2-oxo-4-oxacyclohexyl)hydrazine-1,2-dicarboxylate* (**10b**). Obtained as a colourless oil (0.20 g, 51%). ν_max_(neat) 3272 br, 2965 s, 1798 s and 1716 cm^−1^ s; [α]_D_^20^ -25.0 (c = 0.4, CHCl_3_); δ_H_ 2.4–2.6 (2H, m, CH_2_CO), 3.4–3.6 (2H, m, CH_2_O), 4.1–4.5 (2H, m, CH_2_O), 4.6–5.3 (5H, m, 2 × CH_2_Ph and NCH), 6.5–6.7 (1H, br, NH), 7.2–7.4 (10H, m, ArH); δ_C_ (125 MHz) 25.3 (CH_2_), 42.3 (CH_2_), 64.2 (CH_2_), 67.4 (CH), 67.8 (CH_2_), 67.9 (CH_2_), 128.0 (ArCH), 128.1 (ArCH), 128.2 (ArCH), 128.3 (ArCH), 128.4 (ArCH), 128.5 (ArCH), 135.4 (ArC), 135.6 (ArC), 155.4 (NCO_2_), 155.7 (NCO_2_), 203.4 (CO); m/z(ES^+^) 421 (M+Na^+^, 100), 819 (2M+Na^+^, 85), 1117 (3M+Na^+^, 32); Found (ES^+^) 819.2853, C_42_H_44_N_4_O_12_Na, (2M+Na^+^) requires 819.2812; HPLC (Chiralpak OD-H using hexane: ^i^PrOH (90:10) as solvent at a flow rate of 0.9 mL/min) retention times 37.1 (minor) and 57.3 (major) minutes.

*Dibenzyl (R)-1-(1-methyl-2-oxopropyl)hydrazine-1,2-dicarboxylate* (**10c**) [67,69]. Obtained as colourless needles (0.09 g, 31%). m.p. 59–60 °C; ν_max_(neat) 3255 m, 2958 w, 2884 w, 1749 s, 1703 s and 1512 cm^−1^ m; [α]_D_^20^ -16.0 (c = 0.4, CHCl_3_); δ_H_ 1.36 (3H, d *J* = 16.6 Hz, C*H*_3_CH), 2.01 (3H, s, CH_3_CO), 4.9–5.1 (5H, m, 2xCH_2_ and NCH), 6.6–6.8 (1H, br, NH), 7.1–7.4 (10H, m, ArH); δ_C_ (75MHz): 13.1 (CH_3_), 26.3 (CH_3_), 67.7 (OCH_2_Ph), 67.9 (OCH_2_Ph), 68.5 (NCH), 127.8 (ArCH), 128.0 (ArCH), 128.1 (ArCH), 128.2 (ArCH), 135.7 (ArC), 135.8 (ArC), 156.1 (NCO_2_), 156.4 (NCO_2_), 206.4 (CO); HPLC (Chiralpak OD-H using hexane: ^i^PrOH (93:7) as solvent at a flow rate of 0.9 mL/min) retention times 12.5 (minor) and 15.0 (major) minutes.

## 4. Conclusions

Propylene carbonate is a sustainable and environmentally friendly replacement for dichloromethane and acetonitrile in proline catalysed α-hydrazinations of aldehydes and ketones. Comparable enantioselectivities to those seen with conventional solvents can be obtained, even with a lower catalyst loading. Use of enantiomerically pure propylene carbonate is not advantageous for this reaction.

## Figures and Tables

**Table 1:** Effect of solvent and temperature on proline-catalysed α-hydrazinations of aldehydes.

Entry	Solvent	Aldehyde	Product	Time (h)	T (°C)	Yield (%)	ee (%)
1	CH_2_Cl_2_	**4a**	**7a**	2	RT	86	98 (*R*)^a^
2	**2**	**4a**	**7a**	2	RT	74	69 (*R*)^a^
3	**3**	**4a**	**7a**	2	RT	81	80 (*R*)^a^
4	**3**	**4a**	**7a**	2	0	18	99 (*R*)^a^
5	**3**	**4a**	**7a**	24	0	69	97 (*R*)^a^
6	**3**	**4a**	**7b**	24	0	39^c^	66 (*R*)^b^
7	**3**	**4b**	**7c**	24	0	41	90 (*R*)^d^
8	**3**	**4c**	**7d**	24	0	70	36 (*R*)^d^
9	**3**	**4d**	**7e**	24	0	76	99 (*R*)^a^
10	**3**	**4e**	**7f**	24	0	87	92 (*R*)^a^

a) ee and absolute configuration obtained by chiral HPLC of alcohol **7** [64]. b) ee and absolute configuration obtained by chiral GC of oxazolidinone **8** [65]. c) yield of carbamate **8**. d) ee obtained by chiral HPLC of alcohol **7**, absolute configuration assigned by analogy with other products.

**Table 2:** Proline-catalysed reaction of ketones with dibenzyl azodicarboxylate.

Entry	Solvent	Ketone	Product	Yield (%)	ee (%)
1	Racemic-**3**	**9a**	**10a**	71	77 (*R*)^a^
2	Racemic-**3**	**9b**	**10b**	51	72 (*R*)^b^
3	Racemic-**3**	**9c**	**10c**	31	52 (*R*)^a^
4	(*R*)-**3**	**9a**	**10a**	81	74 (*R*)^a^
5^c^	(*R*)-**3**	**9a**	**10a**	78	75 (*S*)^a^

a) ee and absolute configuration obtained by chiral HPLC [66,67]. b) ee obtained by chiral HPLC, absolute configuration assigned by analogy with product **10a**. c) using (*R*)-proline as catalyst.
